# Differentiation of MS lesions through analysis of microvascular distribution

**DOI:** 10.1162/imag_a_00357

**Published:** 2024-11-08

**Authors:** Linda Sundvall, Irene Klærke Mikkelsen, Simon F. Eskildsen, Mette Madsen Hjørringgaard, Mikkel Nygaard, Peter Vestergaard Rasmussen, Thor Petersen, Leif Østergaard

**Affiliations:** Department of Neurology, Aarhus University Hospital, Aarhus, Denmark; Center of Functionally Integrative Neuroscience (CFIN), Aarhus University, Aarhus, Denmark; Section of Neuroradiology, Department of Radiology, Aarhus University Hospital, Aarhus, Denmark; Neurology Research Unit, University Hospital of Southern Denmark, Aabenraa, Denmark; Department of Regional Health Research, University of Southern Denmark, Odense, Denmark

**Keywords:** multiple sclerosis, white matter injury, perfusion-weighted imaging, capillary transit-time heterogeneity, biophysical tissue oxygenation estimates

## Abstract

Conventional MRI is crucial for diagnosing multiple sclerosis (MS) but lacks precision, leading to the clinico-radiological paradox and misdiagnosis risk, especially when confronted with unspecific lesions not related to MS. Advancements in perfusion-weighted imaging (PWI) with an algorithm designed for diseases with anticipated contrast agent extravasation offer insight into microvascular impairment and flow heterogeneity. Our study aimed to assess these factors in MS patients and their association with clinically relevant white matter injury and disease course. We evaluated 60 adults with white matter lesions (WML), including 50 diagnosed with MS or MS syndromes and 10 non-diseased symptomatic controls (SC) with unspecific WML. MRI included conventional three-dimensional (3D) T2-weighted fluid-attenuated inversion recovery (T2-FLAIR), 3D magnetization-prepared two rapid acquisition gradient-echo (MP2RAGE), post-contrast 3D T1-weighted (T1) images, and Dynamic Susceptibility Contrast (DSC) PWI at 3T. WML masks of “unspecific T2-FLAIR lesions”, “MS T2-FLAIR lesions”, and “MS T1-lesions” were manually outlined and validated by a neuroradiologist. DSC-derived parameters were analyzed in WML masks and healthy-appearing tissue. MS T2-FLAIR lesions showed increased flow heterogeneity and vasodilation compared to unspecific T2-FLAIR lesions in SC, as well as compared to unspecific T2-FLAIR lesions within the MS group. MS T1-lesions exhibited more homogenized flow. Our findings suggest that DSC-PWI, combined with lesion delineation, can provide clinically relevant differentiation of MS lesions from unspecific WML, highlighting potential microvascular pathology previously overlooked in MS.

## 1 Introduction

Magnetic resonance imaging (MRI) plays a critical role in the diagnosis and monitoring of multiple sclerosis (MS) as it can identify blood-brain barrier (BBB) disruption in lesions caused by new inflammation and active disease (Thompson et al., 2018). However, conventional T1-weighted and T2-weighted MRI scans do not correlate strongly with the disease burden, a phenomenon referred to as the clinico-radiological paradox (Mollison et al., 2017). Dichotomizing scans into “positive” or “negative” outcomes can lead to diagnostic delays (Patti et al., 2022) and misdiagnosis (Solomon et al., 2016), with unspecific brain lesions being a significant predictor of misdiagnosis (Wang et al., 2023). Therefore, more advanced MRI methods are necessary to capture the full spectrum of MS disease activity. Longitudinal MRI studies suggest that blood flow changes may precede BBB disruption in lesions and new inflammation (Broom et al., 2005;Hannoun et al., 2015;Wuerfel et al., 2004). Assessing perfusion could, therefore, provide complementary quantitative markers for monitoring or detecting recent/active disease (Ge et al., 2005;Hojjat et al., 2016;Lapointe et al., 2018;Varga et al., 2009).

The concept of microvascular involvement in MS is not new, as early histopathological studies hinted at the presence of hypoxic and ischemic changes in demyelinated lesions (Lassmann, 2003). Previous perfusion studies have investigated BBB integrity in MS pathogenesis (Cramer et al., 2014) and the influence of brain perfusion on both the persistence and repair of MS lesions (Holland et al., 2012). While studies underscore potential microvascular disturbances and oxidative stress in the evolution of MS lesions (Argaw et al., 2012;Geraldes et al., 2017;Haider et al., 2011;Trapp & Stys, 2009), it is noteworthy that MS lesions themselves do not consistently exhibit reduced blood flow (Ge et al., 2005;Peruzzo et al., 2013;Wuerfel et al., 2004), which may seem contradictory. Understanding the relationship between MS lesion progression and hypoxic changes is crucial for identifying specific treatments and interventions to mitigate these effects. Hence, further investigation into alternative microvascular biomarkers is warranted.

When addressing vascular pathology, compromised tissue oxygenation is not necessarily linked to tissue with reduced flow. Tissue oxygenation also depends on the capillary distribution of oxygenated blood (Stefanovic et al., 2008)—and may decrease due to the “shunting” of oxygenated blood that ensues if some capillary transit times become too short to allow efficient oxygen extraction (Angleys et al., 2015;Østergaard et al., 2015). Therefore, perfusion assessments lacking information about capillary transit-time heterogeneity (CTH) may overlook crucial microvascular pathology. Blood’s microvascular distribution can now be estimated through dynamic susceptibility contrast-enhanced MRI (DSC-MRI) by measuring blood’s mean transit time (MTT) as well as the corresponding CTH (Mouridsen et al., 2014;Mouridsen, Christensen, Gyldensted, & Ostergaard, 2006). A biophysical model has been developed to assess tissue oxygenation based on CTH and MTT, in terms of the tissue oxygen tension they provide at normal cerebral oxygen consumption, or*vice versa*(Jespersen & Østergaard, 2012). Moreover, algorithms have been developed to provide reliable CTH and MTT maps despite contrast media leakage, which allow their measurements in conditions that affect the BBB (Hansen et al., 2017).

Efficient oxygen extraction requires a homogenous distribution of blood across capillaries, that is, low CTH. In neurodegenerative/neurovascular diseases, however, high CTH may cause severe tissue hypoxia despite normal CBF. Termed capillary dysfunction, this condition may arise from age-related changes in capillary morphology, vascular comorbidities, inflammation, or angiogenesis (Lauer et al., 2017;Ostergaard et al., 2013;Østergaard et al., 2014). Assessing CTH shows promise in cerebral adrenoleukodystrophy (CALD), an inflammatory demyelinating disease associated with vascular pathology (Lauer et al., 2017). However, the possible application of the DSC biophysical model in MS requires further investigation.

This study aimed to investigate microvascular blood flow heterogeneity in the brains of individuals with MS and determine whether changes in CTH are associated with clinically relevant white matter injury and disease course. Utilizing advancements in DSC methodology, we characterized microvascular perfusion patterns in MS lesions, unspecific white matter lesions (WML), and healthy brain regions of non-diseased symptomatic controls (SC) and individuals newly diagnosed with MS.

## 2 Methods

### 2.1 Materials

A consecutive cohort of patients referred for suspected MS was recruited upon admission to the MS clinic from January 2018 to June 2020. Inclusion criteria comprised patients aged >18 years who had not undergone disease-modifying therapy or received immunosuppressive drugs. All subjects provided informed consent for clinical examination, central nervous system (CNS) MRI examination and were offered analysis of cerebrospinal fluid (CSF). Exclusion criteria encompassed concurrent morbidity, including autoimmune diseases, other neurological disorders, or active cancer, as well as evidence of acute inflammatory processes (leukocyte count >11,000/ml) or contraindications to contrast media injection (eGFR <45 ml/min). In addition, contrast injection was administered only to patients with visible white matter signal abnormalities on T2-weighted MRI due to ethical considerations, leading to the exclusion of subjects without WML.

### 2.2 Clinical data registration

Subjects were allocated into either the MS group or a cohort referred to as non-diseased Symptomatic Controls (SC). Diagnosis of MS and disease courses were conducted in accordance with the 2017 McDonald criteria (Thompson et al., 2018). The SC cohort comprised individuals presenting with neurological symptoms lacking objective clinical or paraclinical findings sufficient to define a specific neurological disease. Note that SC is designated through exclusion criteria and thus, does not represent early MS (Teunissen et al., 2013). Clinical data were collected during the subjects´ initial visit and at the first follow-up approximately 6 months later. The recorded clinical characteristics encompassed the Expanded Disability Status Scale (EDSS), disease course (Clinical Isolated Syndrome CIS, Radiologic Isolated Syndrome RIS, Relapsing-Remitting MS RRMS, Primary-Progressive MS PPMS), along with clinical MRI and CSF markers for MS evaluation. Patients referred due to incidental MRI findings suggestive of MS without typical MS symptoms were diagnosed with Radiologic Isolated Syndrome (RIS). We conducted a diagnostic verification of all patient records at least 2 years after recruitment.

### 2.3 Ethical approval

Written informed consent was obtained from all patients before inclusion. The Central Denmark Region Committee on Research Ethics (no. 1-10-72-180-17) and the Danish Data Protection Agency (no. 2012-58-006) approved the study. The study was registered at ClinicalTrials.gov (no. NCT03906370).

### 2.4 MRI protocol

The MRI protocol included three-dimensional (3D) T2-weighted fluid-attenuated inversion recovery (T2-FLAIR) images and pre-contrast 3D T1 magnetization-prepared 2 rapid gradient-echo (MP2RAGE) images, a DSC scan, as well as post-contrast 3D T1-weighted (T1) images. All images were acquired on a Siemens Magnetom Prisma 3T scanner. SeeSupplementary Methodsfor details on the MR acquisition parameters. The image processing pipeline is shown inFigure 1.

**Fig. 1.**
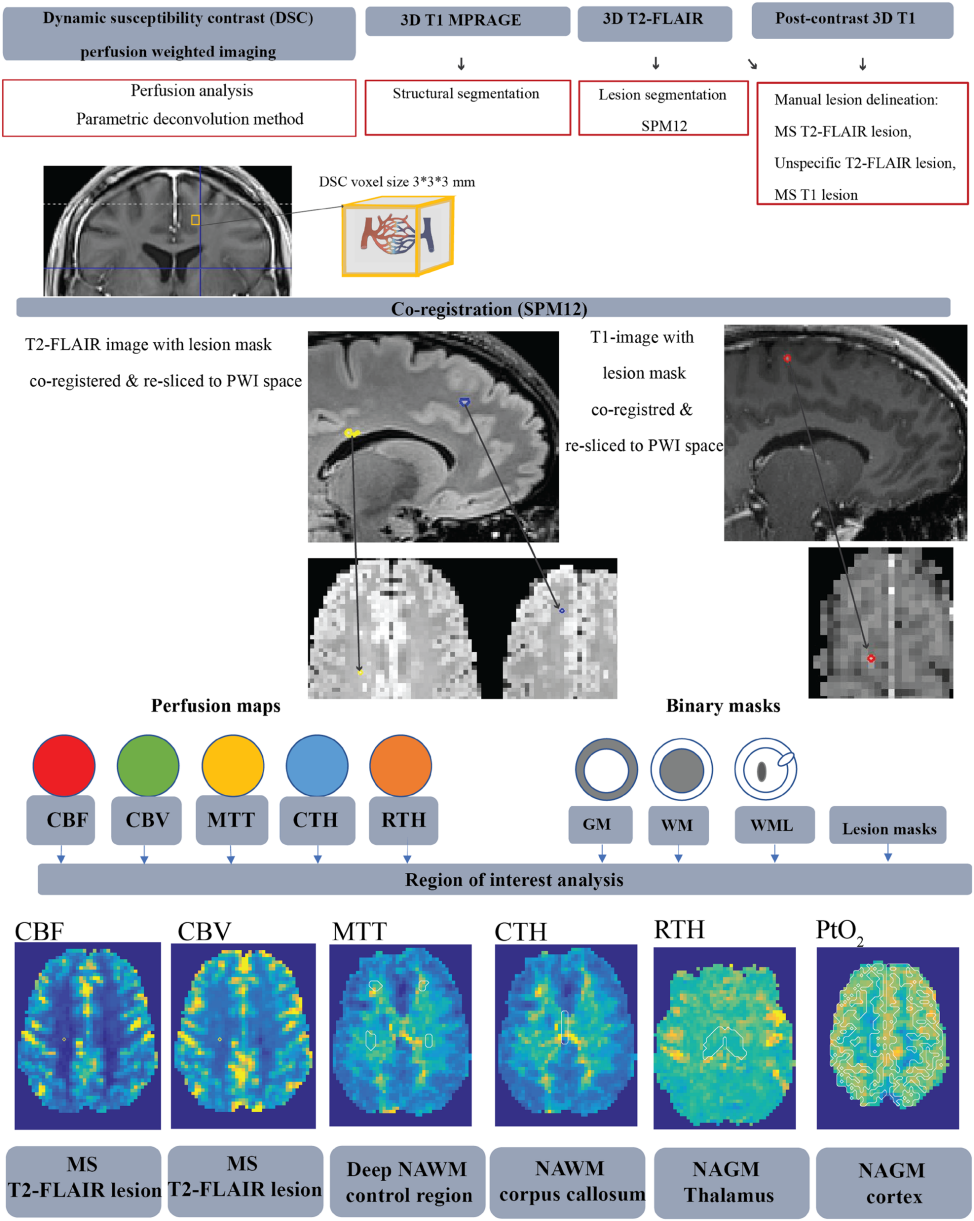
Flow chart of the image processing pipeline. The deconvolution approach applied to the DSC data utilizes a parametric method linking the residue function to the distribution of vascular transit times, h(t), with capillary transit time heterogeneity (CTH). Tissue segmentation generated binary structural masks of gray matter (GM), white matter (WM), and cerebrospinal fluid (CSF). Regional brain volumes (cortex, thalamus, corpus callosum, and deep normal-appearing white matter (NAWM)) were segmented and co-registered to the perfusion maps. Manual delineation of MS lesions and unspecific lesions, along with auto-generated white matter lesion (WML) masks, were employed as exclusion masks to generate NAWM and normal-appearing gray matter (NAGM) compartments. Perfusion parameters were extracted for the manually drawn lesion mask and selected NAWM and NAGM regions. Region of interest (white line). Illustration inspired bySowa et al. (2015).

### 2.5 Investigation of microvascular distribution and brain oxygenation (DSC MRI)

We utilized a methodology adapted from the Centre of Functional Integrative Neuroscience (CFIN) to ensure robust DSC measurements, with the parameters detailed inTable 1.Figure S1andSupplementary Methodsoutline the basic principles of DSC MRI analysis. Briefly, our approach employed an algorithm designed for diseases with anticipated contrast agent extravasation (Hansen et al., 2017), producing leakage-insensitive maps of cerebral perfusion parameters: CBF, cerebral blood volume (CBV), MTT, and CTH. MTT represents the mean transit time of blood through the capillary bed, while CTH reflects the standard deviation of this distribution (Jespersen & Østergaard, 2012). The distribution was modeled using a family of gamma variate functions (Mikkelsen et al., 2015;Mouridsen et al., 2014). Relative heterogeneity of transit times (RTH), calculated as RTH = CTH/MTT, provided insights into deviations from the normal, linear relation between CTH and MTT (Engedal et al., 2018). Additionally, the extended flow-diffusion model allowed the estimation of the tissue oxygen tension (P_t_O_2_), by assuming that hemodynamic parameters sustain the metabolic requirements of resting brain tissue (CMRO2 = 2.5 ml/100 ml/min) (Jespersen & Østergaard, 2012). To maintain the same variance level in the parameter estimates in different regions, the leakage correction was applied in all voxels regardless of contrast enhancement status. To minimize artifacts, voxels with high CBV were excluded. Specifically, the upper 8% of voxels with the highest blood volumes, with an average volume of 113 ml (mean ± 13.1 ml), were removed. This also excluded image voxels consisting only of large blood vessels where the biophysical model is not applicable. The processing pipeline was developed in Matlab (Mathworks Inc).

**Table 1.** Investigated DSC metrics.

	Units	Calculation
rCBF ^a^	Unitless	Deconvolution analysis
rCBV ^a^	Unitless	Central volume theorem
MTT	Seconds	Deconvolution analysis
CTH	Seconds	Deconvolution analysis
RTH	Unitless	Ratio of CTH to MTT
PtO _2_	mmHg	The extended flow-diffusion model

Note:^a^Expressed as relative estimates after normalization to deep NAWM.

### 2.6 Lesion mask delineation

Binary lesion masks were outlined on sagittal T2-FLAIR images and post-contrast sagittal T1 images. Employing intensity thresholding and level sets, multiple masks were manually delineated on each image slice to encompass lesion areas categorized as:

- *MS T2-FLAIR LESIONS*: WML hyperintense on T2-FLAIR but not on post-contrast T1 images, typical appearance of MS lesions.
- *MS T1 LESIONS:*WML hyperintense on post-contrast T1 and T2-FLAIR, typical appearance of MS lesions.
- *UNSPECIFIC T2-FLAIR LESIONS*: punctiform WML hyperintense on T2-FLAIR, without typical appearance of infectious, inflammatory, metabolic, or neoplastic processes, microbleeds, or large ischemic insult. In SC, radiological characterization of “nonspecific pattern” or “micro-vascular pattern” was included (Weidauer et al., 2020). Given the partially overlapping image morphology, lesion interpretation relied on the clinical context, incorporating demographics and clinical characteristics.

Lesion delineation excluded non-relevant signals, motion artifacts, ventricular edge effects, skull, and signal inhomogeneities, confined to lesions within the cerebral hemispheres with a minimum size of three voxels (3.0 x 3.0 mm). MS lesions vary significantly in size, from a few millimeters to several centimeters. The contribution of surrounding tissue to the signal intensity within lesion ROIs depends on both lesion size and the resolution of the MRI sequence in question. We mitigated such partial volume effect (PVE) by requiring lesions to have a cross-section of at least 3 mm (3 pixels) in at least two contiguous slices on T2-FLAIR images during manual segmentation. Moreover, DSC voxels (3 mm in-plane voxel size) containing less than 50% lesion, as determined by this higher-resolution segmentation and nearest neighbor analyses, were omitted from the DSC-parameter analyses. Lesion masks were manually drawn by an experienced operator (LS, 1 year-of-experience) and validated by a certified neuroradiologist (MH, 10 years-of-experience) for accuracy and differential diagnosis. An automatically generated WML mask was created using the Matlab-based lesion segmentation toolbox (LST version toolbox 2.0.15) (Schmidt et al., 2012) and combined with the manually drawn lesion mask for the generation of normal-appearing white matter (NAWM) and normal-appearing gray matter (NAGM) compartments, serving as exclusion masks.

### 2.7 Tissue segmentation

3D T1 MP2RAGE images were segmented into GM, WM, and CSF with an automated classification algorithm to provide compartments of NAWM and NAGM and to accurately identify tissues in specific structures: corpus callosum, thalamus and cortex, frontal and parietal deep white matter, the latter serving as control region (Aubert-Broche et al., 2013;Collins, et al., 1994;Collins & Evans, 1997;Coupe et al., 2008;Coupé et al., 2011;Eskildsen et al., 2012;Naess-Schmidt et al., 2016;Zijdenbos et al., 2002). Find more information on the segmentation methodology inSupplementary Methods.

### 2.8 Region of interest analyses

DSC-derived metrics and biophysical parameters were extracted from lesion masks and NAWM and NAGM compartments for Region of Interest (ROI) analysis. Mean perfusion metrics (CBF, CBV, MTT, CTH, RTH) within each ROI were computed for each subject. In the NAWM and NAGM compartment’s ROI analysis, both the auto-generated WML mask and the manually drawn lesion mask were used as exclusion masks. An NAWM area in the deep frontal and parietal white matter, where MS lesions typically do not manifest, served as a “control region.” We utilized this region to normalize CBF and CBV estimates extracted from other compartments. See theSupplementary Methodsfor further details.

The following ROIs were registered:

- Lesion masks: Unspecific T2-FLAIR lesions, MS T2-FLAIR lesions, MS T1 lesions.
- Corpus callosum NAWM
- Cortical NAGM
- Thalamus NAGM
- Frontal and parietal deep NAWM (ROI used for normalization of CBV and CBF)

### 2.9 Statistics

Each ROI’s perfusion metrics (CBF and CBV) were normalized to the NAWM control region and expressed as relative estimates (rCBF and rCBV). CTH, MTT and RTH were expressed in absolute estimates. Analyses were conducted using STATA 17.0 (StataCorp LLC, Texas, USA). Normality assumptions were assessed through histograms and QQ plots. Continuous data were presented as mean ± SD or median (interquartile range, IQR) and analyzed using paired t-tests or Wilcoxon signed-rank tests for normally and non-normally distributed data. Multiple regression models incorporating potential confounding factors (age, sex) were used to analyze differences in DSC biomarkers between MS and SC, as well as variations across different disease course groups. A two-sided p-value of <0.05 was considered significant.

## 3 Results

We screened 93 patients during the inclusion period. Sixty-one of these met our inclusion criteria with WML detected on MRI. Following comprehensive automated and manual corrections, the dataset comprised 50 MS subjects and 10 non-diseased SC. One subject was excluded due to technical failure.

All MS subjects had cerebral MS T2-FLAIR lesions, with 17 showing enhancing MS T1-lesions and 31 showing additional unspecific T2-FLAIR lesions. ROI analysis was performed for MS T2-FLAIR lesions in 48 MS subjects and enhancing T1-lesions in 16 MS subjects. ROI analysis for unspecific T2-FLAIR lesions was conducted in all 10 SC subjects and 31 MS subjects. None of the unspecific T2-FLAIR lesions in SC showed contrast enhancement or required a change in management. The recruitment overview is inFigure S2.

### 3.1 Clinical characteristics

MS patients and SC cohorts had comparable ages (39.3 vs. 43.3 years), with a slight gender distribution difference, mainly due to more females in the SC group (p = 0.08) (Table 2). Most MS patients had mild disability (mean EDSS 1.6) and RRMS (62%). All SC maintained their non-diseased status over a 2-year period, as confirmed by their electronic health records, with no diagnoses of neurological or autoimmune disease. MS patients exhibited a higher Immunoglobulin G index (0.9 vs. 0.5 index) and a higher prevalence of oligoclonal bands in the CSF (76 vs. 0%) compared to SC. Paraclinical characteristics are shown inSupplementary Table S1.

**Table 2.** Clinical profile of MS and SC.

	No (n)	MS	SC	p-value
Age	50/10	39.32 (10.73)	43.27 (12.28)	0.25
Sex	50/10			0.08
Female		58% (29)	90% (9)	
Neurological complaints				0.34
Opticus neuritis symptoms ^a^		26% (13)	20% (2)	
Sensory ^b^		52% (26)	60% (6)	
Motor ^c^		14% (7)	0% (0)	
Cerebellar ^d^		6% (3)	20% (2)	
Other ^e^		2% (1)	0% (0)	
Time since relapse onset (months)		6.5 (21.5)	-	
EDSS	49/0	1.6 (1.6)	-	
Baseline disease stage				
CIS		10% (5)	-	
RIS		14% (7)	-	
RRMS		62% (31)	-	
PPMS		14% (7)	-	
Enhancing T1-lesion volume (ml) ^f^	16/0	0.43 (0.98)	0	
MS T2-FLAIR lesion volume (ml) ^f^	48/0	2.47 (3.93)	0	
Unspecific T2-FLAIR lesion volume (ml) ^f^	31/10	0.09 (0.07)	0.29 (0.22)	0.001
Unspecific T2-FLAIR lesion classification				
Punctiform, microvascular pattern ^g^		-	50% (5)	
Punctiform, unspecific pattern ^h^		-	50% (5)	

Note:^a^Poor color vision, vision loss, eye pain.^b^Numbness, pain, tingling, burning.^c^Gait change, spasticity.^d^Balance or coordination problems, slurred speech, vertigo.^e^Patient with RIS, without typical MS symptoms.^f^Lesion load based on manually outlined lesion masks co-registered to perfusion maps.^g^The neuroradiologist noted punctiform lesions corresponding to microvascular lesions. Additionally, one patient had an incidental lacune.^h^The neuroradiologist noted an unspecific punctiform pattern. One patient exhibited typical migraine-related signal abnormalities.

#### 3.2.1 Normal CBF but prolonged MTT in MS T2-FLAIR lesions

We noted that although rCBF was not reduced in MS-affected tissue, MTT was prolonged. Noting that CBF equals the CBV:MTT ratio, this was ascribed to elevated CBV in MS-affected tissue, seeFigure 2. Full data tables are in theSupplementary Table S2.

**Fig. 2.**
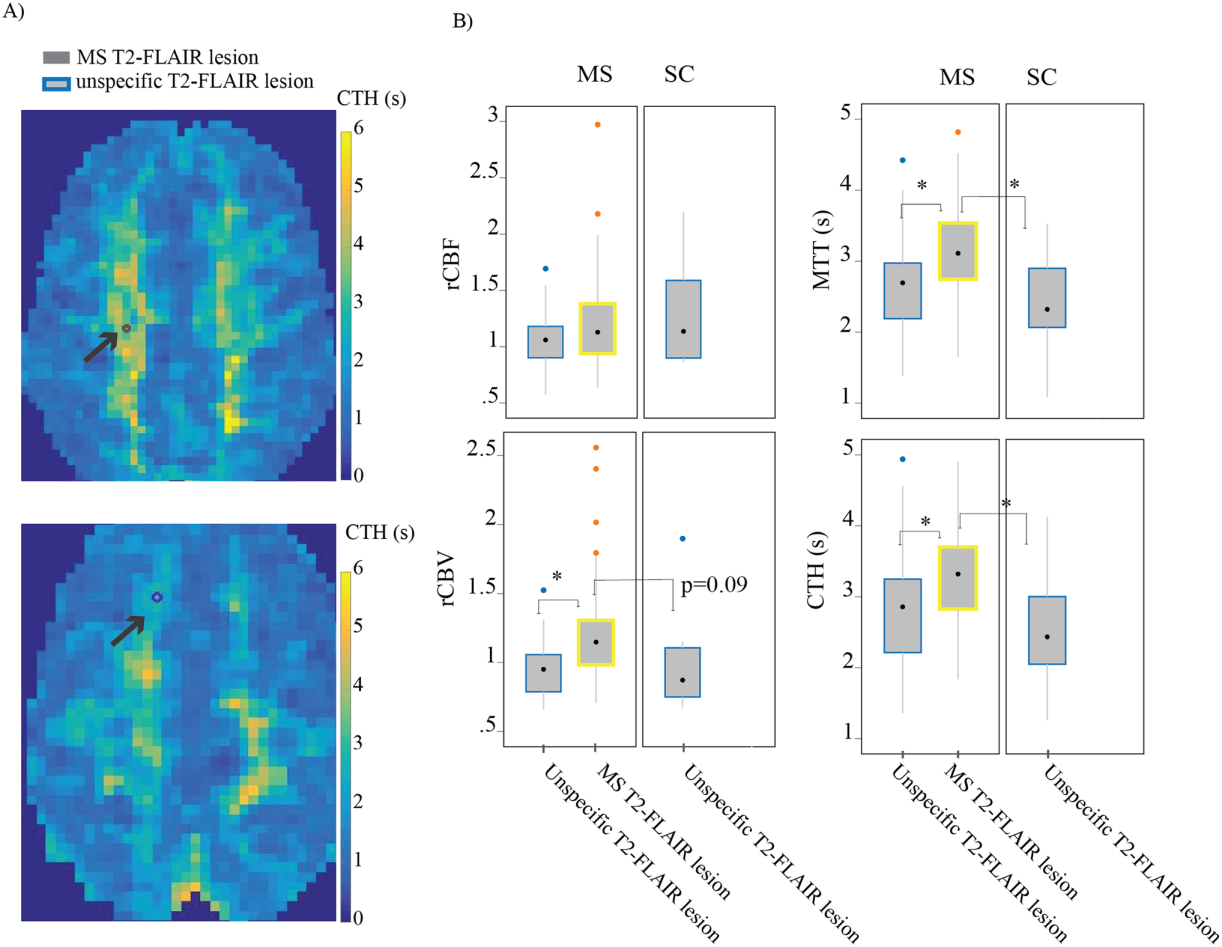
Capillary flow disturbances in MS T2-FLAIR lesions compared with unspecific T2-FLAIR lesion. (A) Example of CTH parametric map with re-sliced T2-FLAIR lesion masks in same patient with RRMS. The grey circle represent the MS T2-FLAIR lesion mask, and the blue circle represent the Unspecific T2-FLAIR lesionmask. (B) Graphs showing MS T2-FLAIR lesions present with increased heterogeneous flow (increased CTH) and indicators of vasodilation (increased rCBV and prolonged MTT) compared to unspecific T2-FLAIR lesions. Data are based on DSC results extracted from MS (pooled across disease course CIR/RIS/RRMS/PPMS) (n = 48) vs. SC (n = 10). The black dot represents the median, and the box between the whiskers indicates the interquartile range (IQR). Outliers, if present, are marked beyond the whiskers. Multivariate regression, controlling for age and sex, tested the hypothesis of no difference between MS and SC. *p-value <0.05.

#### 3.2.2 Blood’s microvascular distribution differs between MS and unspecific T2-FLAIR lesions

MS T2-FLAIR lesions exhibited significantly more heterogeneous flow (high CTH), and prolonged MTT compared to unspecific T2-FLAIR lesions in SC, as well as compared to unspecific T2-FLAIR lesions within the diseased group (Fig. 2). Notably, the deep NAWM selected as the control region was also characterized by high CTH and prolonged MTT when compared to unspecific T2-FLAIR lesions. Additionally, the calculation of PtO2 in extended flow-diffusion model suggested that high CTH and prolonged MTT in MS T2-FLAIR lesions were related to altered tissue oxygenation.Table 3summarizes the overall perfusion changes in T2-FLAIR lesions compared to NAWM control region.

**Table 3.** T2-lesion’s perfusion characteristics when compared with NAWM.

	rCBV	rCBF	RTH	CTH	MTT	PtO _2_
MS T2-FLAIR lesion (n = 48)	↑	↑	↓	↑	↑	↓
Unspecific T2-FLAIR lesion in MS (n = 31)	~	~	↓	↓	↓	↑
Unspecific T2-FLAIR lesion in SC (n = 10)	~	~	~	↓	↓	↑

### 3.3 MS enhancing T1 lesions are characterized by uniform blood distribution

**Fig. 3.**
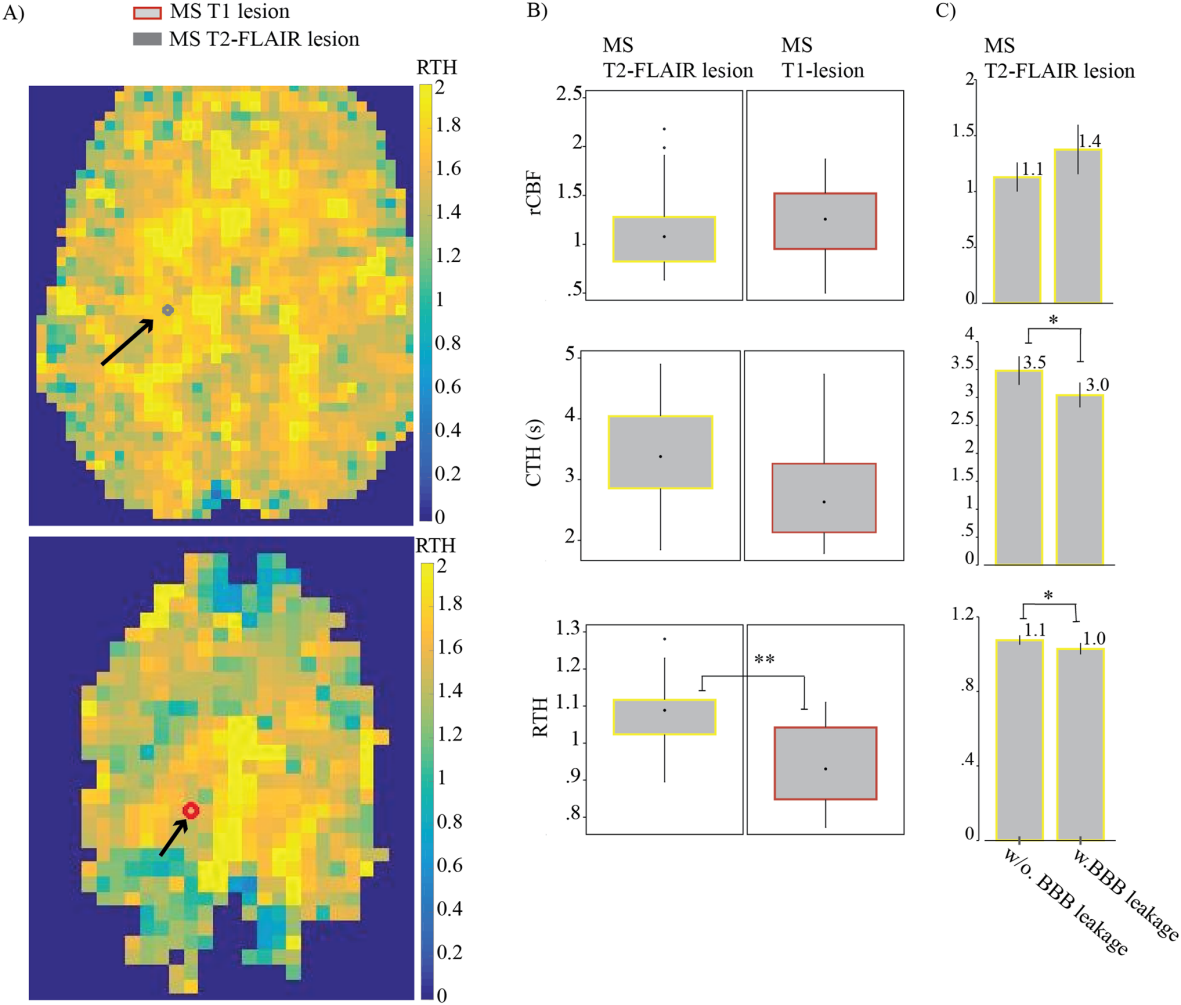
MS T1 lesions exhibit uniform blood distribution. (A) RTH parametric map example with resliced T1- and T2-FLAIR lesion masks in a patient with RRMS. The grey circle represent the MS T2-FLAIR lesion mask, and the red circle represent the T1 lesion mask. (B) Perfusion estimates from MS T1 and T2-FLAIR lesions within individuals with BBB leakage (n = 16). The black dot represents the median, and the box between the whiskers indicates the interquartile range (IQR). (C) Mean perfusion estimates from MS T2-FLAIR lesions stratified by BBB leakage status (with (w) vs. without (w/o) BBB leakage n = 16 vs. n = 32) Vertical line, on top of the bar, to indicate the 95% confidence interval. Homogenization of capillary flow, reducing relative transit time heterogeneity (RTH = CTH/MTT), is crucial for optimal oxygen extraction during increased metabolic demands. The reduced RTH associated with BBB leakage may indicate either active redistribution of blood to meet metabolic needs in inflamed areas and/or a reduction in number of open capillaries due to edema. *p < 0.05.

In MS patients with enhancing T1 lesions (n = 16), capillary flow patterns showed relatively homogenized characteristics, reflected by low RTH compared to their T2-FLAIR lesions (Fig. 3). Note that RTH, the CTH: MTT ratio, is calculated in each image voxel before averaging within and across subject. This explains why significant RTH differences may not be discernable from parallel, averaged CTH and MTT values. The normal CTH: MTT ratio is close to 1.

Despite excluding voxels containing T2-FLAIR and T1 lesion mask overlaps, not all lesions categorized by their T1- and T2-weighted imaging characteristics exhibited similar perfusion alterations. Stratifying subjects based on the presence of enhancing T1 lesions suggested two groups of MS T2 FLAIR lesions: those from MS subjects with simultaneous presentation of T1 lesions (w/ BBB leakage) (n = 16) and those without (w/o BBB leakage) (n = 32). In subjects with BBB leakage elsewhere, MS T2-FLAIR lesions exhibited similar characteristics to T1 MS lesions, including high rCBF and homogenized flow (decreased RTH) due to low CTH relative to MTT.Table 4summarizes how perfusion characteristics of MS lesions vary with BBB Leakage. Full data tables are available inSupplementary Table S3.

**Table 4.** MS lesions perfusion characteristics in subjects with BBB leakage (n = 16).

	rCBV	rCBF	RTH	CTH	MTT	PtO _2_
MS T1-lesion vs. MS T2-FLAIR lesion						
	~	~	↓	~	~	~
MS T1-lesion vs. NAWM						
	↑	↑	↓	~	~	~
MS T2-lesion vs. NAWM						
	↑	↑	↓	~	~	~
MS T2-lesion vs. MS T2-FLAIR lesions in subjects without BBB leakage ^a^						
	~	~	↓	↓	~	~

Note:^a^Difference between MS T2-FLAIR lesions in subjects with/without BBB leakage (n = 16 vs. n = 32).

### 3.4 Capillary dysfunction and vasodilatation are associated with progressive disease course

**Fig. 4.**
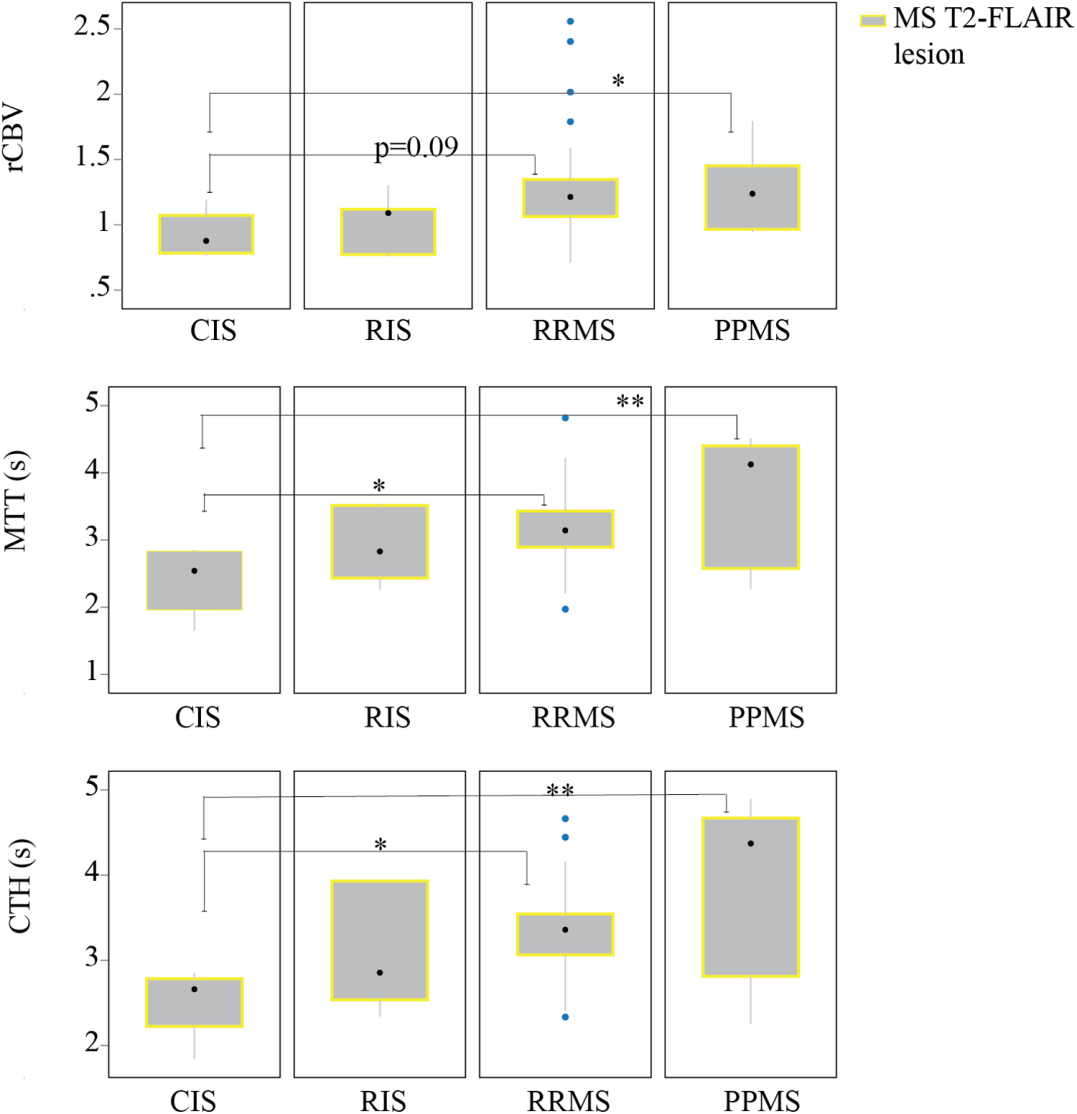
Capillary dysfunction and vasodilation is associated with progressive disease course. Graphs illustrate perfusion differences in MS T2-FLAIR lesions between CIS/RRMS and CIS/PPMS patients. The findings suggest accumulated capillary flow disturbances (high CTH), along with indications of vasodilation (increased rCBV and prolonged MTT) at the progressive disease course. Data are derived from DSC results obtained from MS T2-FLAIR lesion in CIS, RIS, RRMS, and PPMS n = 4/7/30/7. The black dot represents the median, and the box between the whiskers indicates the interquartile range (IQR). Outliers, if present, are marked beyond the whiskers. Multivariate regression, controlling for age, sex, T2-FLAIR lesion volume tested the hypothesis of no difference across disease course. *p-value < 0.05, **p-value ≤ 0.01, ***p-value ≤ 0.001.

We assessed the relationship between MS T2-FLAIR lesion perfusion characteristics and disease course, adjusting for age, sex, and T2-FLAIR lesion volume. The results revealed significant differences in T2-FLAIR lesions between CIS and RRMS patients and between CIS patients and PPMS, indicating elevated CTH, prolonged MTT, and a trend toward increased rCBV in individuals with the progressive disease course (Fig. 4). Full data tables are available inSupplementary Table S4.

### 3.5 Identical perfusion in MS NAWM and NAGM

No perfusion changes were observed in NAWM and NAGM compartments between the MS and SC (Supplementary Fig. S3). Flow patterns varied across regions, with lower CTH in NAGM compared to deep NAWM compartments selected as the control region, indicating an inverse correlation between perfusion and capillary flow heterogeneity. Analysis revealed significant age-related differences in NAGM and significant sex-related differences in NAGM and NAWM. No volume differences were detected between MS patients and SC.

## 4 Discussion

Our investigation into newly diagnosed MS patients revealed that the CTH parameter is sensitive to clinically relevant white matter injury, suggesting advanced DSC perfusion measurements may prove valuable for lesion segmentation and clinical monitoring tasks.

Analysis of MS T2-FLAIR lesions revealed distinct features indicative of altered tissue oxygenation, including heterogeneous flow (high CTH) and markers of vasodilation (prolonged MTT) or potential angiogenesis (increased rCBV). Tissue hypoxia due to reduced blood flow typically shows prolonged MTT and increased CBV, indicating vasodilation, allowing extra time for oxygen diffusion in capillaries with reduced flow. In that context, prolonged MTT often results in a broader distribution, leading to a “passive” increase in CTH (Engedal et al., 2018). In MS T2-FLAIR lesions, increased CTH under normal CBF does not reflect the passive increase observed with a primary reduction in blood flow. Instead, the more heterogenous flow in comparison to other unspecific subcortical white matter lesions, suggests changes in capillary function or patency, leading to a high degree of microvascular shunting. Thus, the risk of compromised tissue oxygenation in MS demyelinated lesions would not be due to limited blood supply, but rather to inefficient oxygen extraction from the microcirculation (Ostergaard et al., 2013;Østergaard et al., 2015).

The correlation between capillary flow disturbances and disease course phenotype unveils a noteworthy trend: cumulative changes in CTH in individuals with progressive disease course. Although relapsing MS often transitions into a progressive stage, PPMS is not necessarily a more advanced form of MS. Biologically, however, our results support the view of a continuum between relapsing and progressive stages, distinguished by quantitative differences in pathological change rather than representing entirely separate diseases. As MS advances and more lesions evolve, this may lead to increasingly compromised brain tissue oxygenation. A longitudinal DSC study on a hereditary demyelinating disease indicated that CTH could predict lesion progression (Lauer et al., 2017), further supporting the notion that more heterogeneous flow may be an indicator of progressive white matter damage associated with demyelination. Additionally, the observed elevation in DSC-measured rCBV in T2-FLAIR lesions aligns with its sensitivity as a marker for neovascularization in brain tumor angiogenesis (Law et al., 2003), potentially extending its applicability to neo-angiogenesis or post-inflammatory vasodilation in MS brain lesions (Gentile et al., 2022).

Identifying imaging biomarkers that differentiate MS lesions from other white matter disorders is challenging and impacts treatment strategies. In our study, high CTH and vasodilation offered clinically relevant differentiation between MS T2-FLAIR lesions and unspecific T2-FLAIR lesions. However, changes in CTH and compensatory vasodilation are not specific to MS and likely mirror a broader hemodynamic pattern in vascular-associated white matter disorders (Dewey et al., 2021). The pathological significance of the unspecific lesions also remains debated, as they can reflect chronic hypoperfusion, small vessel disease, or migraines (Weidauer et al., 2020).

### 4.1 Hemodynamic pattern related to contrast enhancement

Combined with lesion delineation, DSC metrics may serve as an adjunctive tool for lesion evaluation and may potentially also aid in distinguishing between active and chronic lesions. A distinct pattern emerged in enhancing T1 lesions compared to MS T2-FLAIR lesions lacking radiological indications of BBB leakage, as the former demonstrate more uniform microscopic distribution of blood (low RTH). The causes and possible implications of this finding remain unclear. Biophysically, homogenization of microvascular flows (low RTH and/or low CTH) is beneficial in that it improves net oxygen extraction from the available blood supply (Jespersen & Østergaard, 2012) and thus may help cover the metabolic demands of ongoing, inflammatory processes that accompany BBB damage. On the other hand, findings in cerebral ischemia suggest that low RTH also emerges when widespread capillary occlusions affect long, high-resistance capillary paths, severely reducing oxygen extraction despite more homogenous flows among the remaining, open capillaries (Engedal et al., 2018). In our data, homogenization was not accompanied by reductions in relative blood volume, contradicting capillary occlusions as an underlying cause of the homogenization. Importantly, previous studies have even demonstrated increases in blood flow that precede (Broom et al., 2005;Wuerfel et al., 2004) or coincide with the onset of MS T1 lesions (Hannoun et al., 2015). Furthermore, research has identified an enhanced CBF response to neuroactivation during disease exacerbation (Tekgöl Uzuner & Uzuner, 2016;Vestergaard et al., 2022). These findings suggest that active hemodynamic responses play a role in MS neuroinflammation, particularly during episodes of BBB leakage.

### 4.2 Methodological challenges for perfusion-based biomarkers in MS

While perfusion imaging studies have provided valuable insights into MS disease dynamics (Ge et al., 2005;Hojjat et al., 2016;Lapointe et al., 2018;Varga et al., 2009), discrepancies remain in reported perfusion changes. A standardized approach for DSC MRI acquisition and postprocessing is needed to address these inconsistencies. Other studies have reported decreased rCBF in MS T2-FLAIR lesions, with or without prolonged MTT (Ge et al., 2005;Sowa et al., 2015) contrasting with our findings. Additionally, findings regarding deviations in CBF or CBV in acute MS T1 lesions compared to MS T2-FLAIR lesions and NAWM vary across studies (Ge et al., 2005;Hannoun et al., 2015;Sowa et al., 2015;Wuerfel et al., 2004), possibly reflecting technical challenges in perfusion assessment in diseases with contrast agent extravasation and tissue heterogeneities. Previous studies on NAWM and NAGM in MS brains have suggested hypo-perfusion as a potential MS predictor (Adhya et al., 2006;Ge et al., 2005;Hojjat et al., 2016;Rashid et al., 2004;Varga et al., 2009), but differences in disease stages, treatments, and control groups hinder comparisons. Methodological design further complicates interpretations. DSC measurements provide quantitative MTT, CTH, RTH and PtO2 estimates that can be compared across tissue regions and across subjects, whereas CBV and CBF are measured in arbitrary units and typically normalized to NAWM values. Given that white matter lesions may affect DSC parameters in NAWM (Dalby et al., 2019), the normalization we applied to CBV and CBF may be suboptimal. We normalized CBF and CBV measurements to a defined NAWM region for all subjects, and differing NAWM pathology rates between MS and SC may have influenced results. Future studies could include quantitative CBV and CBF methods to quantify white matter hemodynamics, such as PET.

Nonetheless, our perfusion measurements in the normal-appearing brain of MS individuals align with prior investigations on healthy subjects without brain atrophy (Engedal et al., 2018;Nielsen et al., 2017), suggesting reconsidering the prevailing understanding of MS-related hypoperfusion. To enhance the reliability and comparability of DSC studies in MS, we recommend implementing leakage-corrected DSC maps, excluding signals from large blood vessels, manually segmenting lesions, and accounting for demographic variables to minimize confounding. Leakage correction, essential in MS, was performed using a method validated in tumor patients which display far greater levels of contrast leakage than MS patients. Although the assumptions of the leakage correction method appear applicable to low levels of leakage, this requires further experimental validation.

### 4.3 Capillary flow disturbances as a component of MS lesions

Capillary flow disturbances limit oxygen extraction from the bloodstream and are therefore expected to affect the CBF needed to meet local energy demands—keeping in mind that net oxygen equals CBF multiplied by the local oxygen extraction fraction (OEF). Biophysical models predict that*increased*CBF is needed to meet metabolic demands while preserving physiological PtO2 for*mild*capillary flow disturbances, whereas*attenuation*of resting CBF and CBF responses is needed to limit the capillary “shunting” of oxygenated blood that accompanies more*severe*capillary flow disturbances (Angleys et al., 2015;Jespersen & Østergaard, 2012;Østergaard et al., 2013). In the latter case, OEF is predicted to increase while PtO2 falls below physiological levels. As a result, severe capillary flow disturbances may ultimately limit tissue oxygen metabolism and cause hypoxic tissue injury. Studies of CBF changes during functional activation have reported significantly increased and reduced CBF responses, respectively, in MS patients at the individual and group level (Vestergaard et al., 2022). While some of these changes may reflect altered neural network activities, our findings suggest that the degree of capillary flow disturbances in patients’ respective lesions also affects local neurovascular coupling, that is, the CBF response elicited by a certain network activity. We, therefore, propose that capillary flow disturbances should be factored in when correlating CBF- or blood oxygen level-dependent (BOLD) based neural network activity measurements to patients’ symptoms (Angleys et al., 2018).

Studies of cerebrovascular reserve capacity (CVR) in MS show either preserved or reduced CBF responses to vasodilator (e.g. CO_2_) administration, possibly related to disease duration (Marshall et al., 2014;Vestergaard et al., 2022). Recent human data (Aamand et al., 2024) confirm biophysical model predictions that vasodilator responses must be attenuated in order to meet (resting) metabolic demands in the presence of capillary flow disturbances. Future studies should, therefore, address whether reductions in CVR in MS are linked to capillary flow disturbances. Notably,Chiarelli et al. (2022)found that immunomodulation*restores*CVR in MS patients. This observation could relate to reversals of capillary flow disturbances, similar to those observed in other conditions characterized by neuroinflammation, demyelination, and BBB damage (Lauer et al., 2017,^2023^).

Studies show that brain oxygen metabolism is reduced in MS patients (Chandler et al., 2023;Ge et al., 2012) but it is unclear whether these findings reflect impaired oxygen extraction due to capillary flow disturbances, reduced metabolic demands due to other disease changes, or both. We combined DSC measurements and biophysical models to estimate PtO2under the assumption of*normal*oxygen utilization, rather than measuring oxygen metabolism itself. Applying our method to patients with*subnormal*oxygen utilization, our method will, therefore, yield subnormal PtO2 estimates, although the measured hemodynamics, in fact, cover the lower metabolic rate at normal PtO2. Longitudinal studies of both capillary flow disturbances*and*oxygen metabolism are needed to further address links between microvascular changes and neurodegeneration, such as those observed in Alzheimer’s disease (Nielsen et al., 2017). Notably,Chandler et al. (2023)identified widespread reduction of oxygen utilization and the effective diffusivity of the capillary network. The latter is the biophysical hallmark of impaired oxygen extraction caused by capillary flow disturbances and the methodological approach used by Chandler et al. could thus advance our understanding of oxygen transport in MS and the source of the apparent energy crisis that characterizes the disease (Park & Choi, 2020).

The causes of the apparent, MS-related capillary flow disturbances are unclear. Possible sources may include endothelial or perivascular cell damage, impaired pericyte function (Dalkara et al., 2011,^2024^), altered leukocyte adhesion during their capillary passage (e.g., stalled capillary flow) (Lauer et al., 2017), edema-induced capillary compression (Østergaard et al., 2014), and irregular capillary diameters due to angiogenesis (Ostergaard et al., 2013). Products of oxidative damage are prevalent in MS brains (Haider et al., 2011) and associated with meningeal inflammation, BBB disruption (Argaw et al., 2012), and demyelination (Trapp & Stys, 2009). Moreover, hypoxic and inflammatory stimuli can trigger metabolic responses in perivascular cells, leading to vasoactive peptide production, upregulation of genes that promote vessel plasticity and permeability, or pericyte damage (Argaw et al., 2012;Dalkara et al., 2024).

### 4.4 Limitations

As our study’s statistical power is constrained by the relatively small sample size (owing to COVID-related shut-downs), we advocate for further research to employ recommended DSC practices in larger cohorts, thereby advancing the clinical applications of DSC perfusion maps in MS. The low number of recruited non-diseased SC reflects the infrequent occurrence of WML in the investigated age group. Nonetheless, our study’s strengths include adjusting perfusion results for age and sex and the inclusion of a diverse cohort of untreated MS patients. Contrasting MS patients with symptomatic controls, rather than healthy volunteers, enhances the likelihood of identifying abnormalities specifically associated with MS pathology, given the overlap of symptoms and findings often observed in MS.

The difference in MRI voxel sizes between T2-FLAIR (1 mm) and DSC (3 mm) imaging introduces PVE when calculating parameter averages within lesion ROIs transferred to DSC maps. This effect is important given that up to 50% of a lesion may consist of NAWM. Consequently, PVE can diminish the contrast between lesion and NAWM in DSC-derived parameter values. Notably, PVE is more likely to bias parameter averages for smaller lesions, which could affect DSC-parameter average differences between MS lesions and unspecific lesions, as the latter are typically smaller than the former. To mitigate PVE, we excluded lesions smaller than 3 mm across. Future studies should consider using higher-resolution DSC MRI to further reduce the impact of PVE.

## 5 Conclusion

In conclusion, our use of methodological advancements in DSC imaging among newly diagnosed MS patients reveals a distinct pattern of microvascular impairments within MS lesions, offering insights into clinically relevant differentiation from non-specific white matter abnormalities. The observed flow heterogeneity and vasodilation associated with MS may signify an overlooked microvascular pathology in MS lesions, and potentially contribute to the clinico-radiological paradox. Integrating advanced DSC imaging with T1-weighted and T2-weighted MRI shows promise for enhancing our understanding of white matter abnormalities and facilitating algorithm development to predict MS outcomes.

## Supplementary Material

Supplementary Material

## Data Availability

The methodology used for DSC measurements is integrated into CFIN’s imaging databases and is not available as a standalone version. While we are open to sharing code for analyses as part of academic collaborations, we cannot provide support for broader distribution. For those interested in software based on the vascular model, we recommend contacting Cercare Medical.

## Author Contrubtions

L.S.: Investigation, formal analysis, visualization, and writing—Original draft. I.K.M.: Investigation, methodology, validation, supervision, and writing—review & editing. S.F.E.: Methodology, validation, and writing—review & editing. M.M.H.: Investigation, validation, and writing—review & editing. M.N.: Methodology, validation, and writing—review & editing. P.V.R.: Resources, writing—review & editing. T.P.: Conceptualization, resources, supervision, and writing—review & editing. L.Ø.: Conceptualization, resources, methodology, supervision, and writing—review & editing.
