# Supplementary material for: Differentiation of MS lesions through analysis of microvascular distribution
Source: Imaging Neurosci (Camb). 2024 Nov 8;2:imag-2-00357. doi: 10.1162/imag_a_00357 (PMC12290577; doi:10.1162/imag_a_00357)
Supplement: Supplementary Material [file imag_a_00357-supp.pdf]

Supplementary Material

Supplementary methods

1. MRI protocol
2. MR post-processing
3. Parameters on microvascular distribution and brain oxygenation

1. MRI protocol

The MRI protocol included three-dimensional (3D) T2-weighted fluid-attenuated inversion recovery (T2-FLAIR) images and post-contrast 3D T1-weighted (T1) images for lesion outlining, a gradient-echo dynamic susceptibility contrast (DSC) scan for perfusion and brain oxygenation assessment as well as pre-contrast 3D T1 magnetization-prepared 2 rapid gradient-echo (MP2RAGE) for detailed structural assessment and tissue segmentation. All MR images were acquired on the Siemens 3T Prisma scanner;

Pre-contrast sagittal 3D T2-FLAIR images for T2-FLAIR lesion outlining were acquired in 176 slices, TE = 388 ms, TI= 1800 ms, TR = 500 ms, no slice gap, transverse slice orientation, 1 × 1 × 1 mm^3 voxel, 120° refocusing angle.

Pre-contrast 3D T1 MP2RAGE images for tissue segmentation into gray matter (GM), white matter (WM), cerebrospinal fluid (CSF) and white matter lesion (WML) were acquired with 192 slices, TE = 3.47 ms, TI_1= 500 ms, TI_2= 2900 ms, TR = 6500 ms, no slice gap, transverse slice orientation, 0.9 × 0.9 × 0.9 mm^3 voxel, 120° refocusing angle.

DSC perfusion sequence was a gradient echo EPI sequence acquired with 42 axial slices, TE = 32 ms, TR = 0.8s, flip angle 45 or 53 degrees, no slice gap, transverse slice orientation, 3.0 × 3.0 × 3.0 mm^3 voxel in 64x64 matrix. The paramagnetic contrast agent Gadovist (1M, Bayer) was administered with 0.1mmol pr kg body weight with a power-injector at injection rate 5ml/s and followed by a 30 ml saline flush. Injection commenced 15 seconds after DSC imaging onset to provide a baseline prior to contrast arrival to the brain, and bolus duration was standardized to 60 seconds after contrast arrival.

Post-contrast sagittal 3D T1 was acquired for outlining enhanced T1 lesions. The imaging parameters were 176 slices, TE = 2.52 ms, TI= 900 ms, TR = 1900 ms, no slice gap, transverse slice orientation, 1 × 1 × 1 mm^3 voxel, 9° flip angle.

2. MR post-processing

A) Generation of parametric perfusion maps: All dynamically imaged volumes of the DSC sequence were co-registered to the last baseline volume to correct for motion during bolus acquisition. Slice-timing correction was performed, and concentration was calculated using the exponential relation between MR signal changes in the relaxation rate that occurs as the contrast agent passes the vasculature. Figure S1 illustrates the basic principles of DSC MRI analysis.

The contrast concentration curve representing the arterial supply (arterial input function) was automatically detected among voxels with the narrow and early arrival of the contrast agent and with a fast initial increase in area under the concentration curve. Spatially, the search was confined to the region supplied by the middle cerebral artery. Large vessels and CSF were excluded, and the images were smoothed using a Gaussian 3x3 voxels in-plane filter. No temporal smoothing was performed. The tissue concentration curves were corrected for the dispersion of contrast already present in the arterial supply in a deconvolution process. The tissue curves after correction describe the remaining concentration in the capillary web had the bolus been injected instantly into the tissue. The deconvolution strategy was based on a parametric method by Mouridsen et al. and directly relates the residue function R(t) to the distribution of vascular transit times, h(t), and hence to CTH. This residue function is modeled by a family of gamma variate functions (Mouridsen et al., 2014). Specifically, the model includes the delay between supplying artery and tissue curves, the cerebral blood flow, and two model parameters, alpha and beta, describing the shape of the corrected tissue curves. The parameters were fitted using an expectation-maximization fitting algorithm. The starting guess of the parameters was estimated using singular value decomposition. MTT and CTH, being the mean and standard deviation of the distribution of capillary transit times, are given by the gamma variate function. The deconvolution method yields CBF×R(t), the height of which is CBF because R(t) quantifies the fraction of contrast media retained in the microcirculation (Fig. S1). CBV corresponds to the area under CBF×R(t) curve, following the central volume theorem (CBV=CBF*MTT). Using MTT and CTH estimates, a biophysical model by Jespersen and Oestergaard was used to calculate corresponding tissue oxygen pressure (ptO2) (Jespersen & Østergaard, 2012).

Our implementation of CTH estimation originates in the PGUI program, widely used in stroke imaging. The automatic implementation provides parameter estimates within the range of manual operators (Mouridsen, Christensen, Gyldensted, & Ostergaard, 2006; Østergaard et al., 2014). The software has continuously been refined, including a Bayesian approach to perfusion processing. This method estimates CTH more robustly than singular value decomposition (Mouridsen et al., 2014) with added robustness in MTT estimates to bolus duration, as shown in CT imaging of a stroke population (Mikkelsen et al., 2015). This software has been applied in various clinical contexts, including stroke, tumors, CADASIL, and Alzheimer's disease, demonstrating sensitivity to differentiate patient groups.

B) Tissue segmentation: T_1w images were processed using a fully automatic pipeline(Aubert-Broche et al., 2013). Images were denoised(Coupe et al., 2008), registered to MNI space using a 12-parameter affine transformation(Collins, Neelin, Peters, & Evans, 1994) and skull stripped(Eskildsen et al., 2012). Regional brain volumes (i.e., hippocampus, caudate nucleus, putamen, globus pallidus, thalamus, and corpus callosum) were segmented using a patch-based label-fusion method(Coupé et al., 2011), applying manually crafted training data from the same scanner, which has been shown to improve accuracy compared to other methods(Naess-Schmidt

et al., 2016). Tissue segmentation was performed to provide binary structural masks of GM, WM, and CSF using an automated neuronal network classification (Zijdenbos, Forghani, & Evans, 2002) combined with tissue priors non-linearly warped from MNI space to the individual images (Collins & Evans, 1997). The mid-sagittal segmentation of corpus callosum was dilated in the lateral direction to achieve a thickness of 3 mm. Additionally, axial T1 images were employed to manually define regions in the frontal and parietal deep white matter in each hemisphere, designated as control regions for the subsequent region of interest analysis.

C) T2-FLAIR lesion masks and enhancing T1-lesion masks were manually drawn on sagittal 3D T2-FLAIR and post-contrast sagittal 3D T1 images, respectively. An auto-generated non-modified WML mask was segmented by the lesion growth algorithm(Schmidt et al., 2012) as implemented in the LST version toolbox 2.0.15 (www.statisticalmodelling.de/lst.html) implemented in Statistical Parametric Mapping toolbox (SPM12) running in MATLAB R2016b (MathWorks, Natick, MA, USA).

D) Co-registration: Manually drawn lesion masks, auto generated structural masks, and structural 3D T1 MP2RAGE and 3D T2-FLAIR sequences were co-registered and resliced to the average DSC volume using SPM12.

E) Region-of-interest (ROI) analysis. ROI analysis involved extracting DSC metrics from both manually drawn lesion masks and auto generated masks of normal-appearing white matter (NAWM) and normal-appearing gray matter (NAGM) in specific structures (corpus callosum, deep white matter, thalamus, and cortex). All ROIs were excluded of CSF and large vessels. In ROI analysis for NAWM/ NAGM compartments, the WM/GM mask was used as the inclusion mask and auto-generated WML mask, and the manually drawn lesion mask were used as exclusion masks. In ROI analysis, T2-FLAIR-lesions partly overlapping with T1-lesion masks were excluded. Due to the spatial resolution constraint of DSC (3.0 x 3.0 x 3.0 mm^3), we refrained from comparing ring and nodular enhancing T1-lesion patterns. Additionally, no lesion-by-lesion analysis was conducted.

3. Microvascular distribution parameters

According to the classical flow-diffusion equation, hyperemia or increased CBF always results in increased tissue oxygenation. However, according to the extended flow-diffusion model, the upper limit of oxygen availability is a function of CTH at a given blood flow. In Figure S1, the upper curve follows the original flow diffusion equation with low CTH and predicts that oxygen availability varies uniformly as a function of CBF, assuming that capillary flow velocities and length are identical. The lower curve is based on the extended flow-diffusion model, where the extraction of oxygen becomes increasingly inefficient as the capillary flow becomes more heterogeneous. By the extended flow-diffusion model, we can attain surrogate estimates on tissue oxygen tension or the metabolic rate of oxygen, allowing for quantifying dynamic changes in brain oxygenation under both normal and pathological conditions.

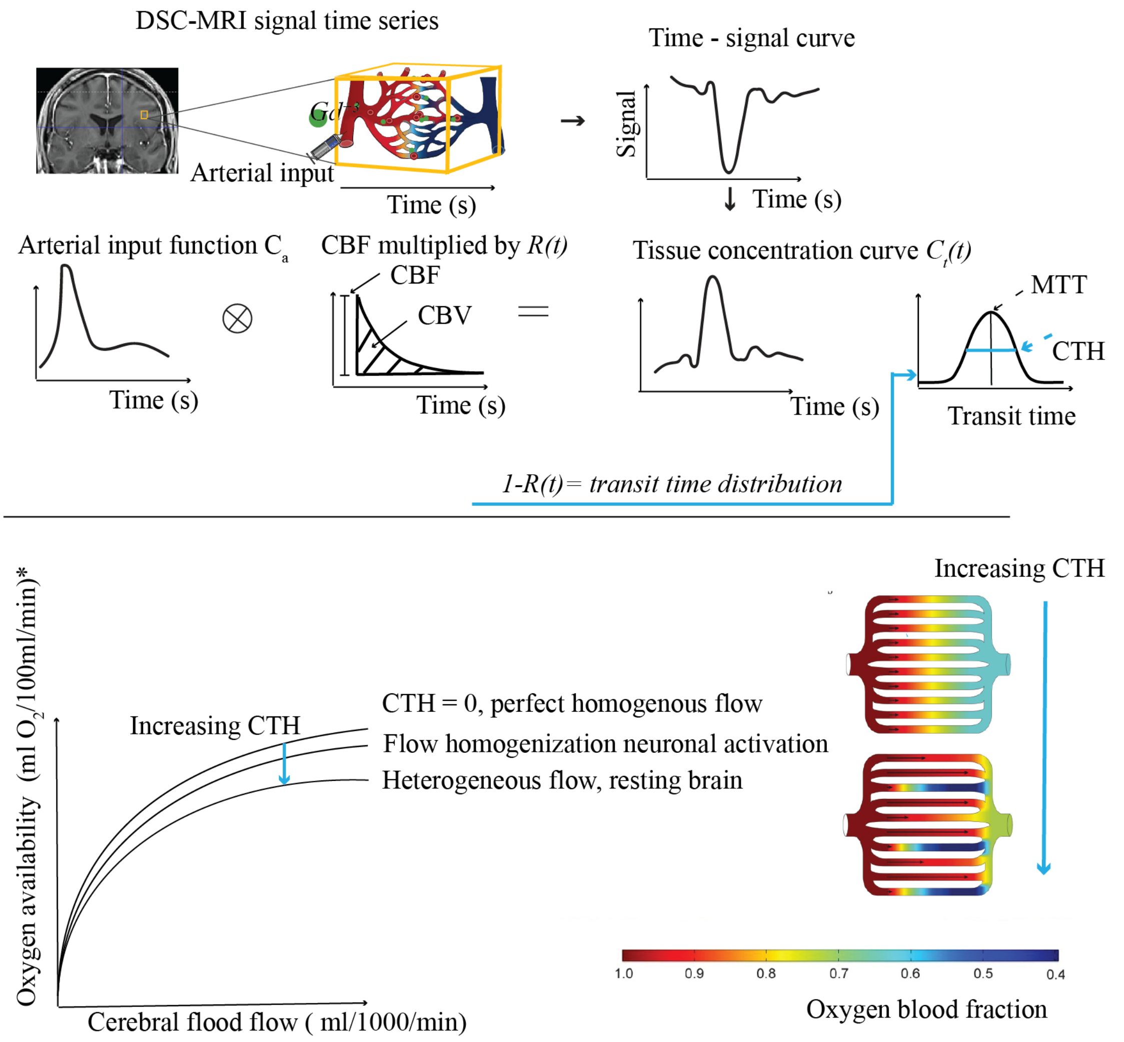

Figure S1. Basic principles of DSC MRI analysis (on top) and graphical presentation of the extended diffusion model (below). Estimation of CTH and tissue oxygenation are based extended classical flow-diffusion theory. For generation of hemodynamic parameters, the change in MRI signal caused by the passage of contrast-agent is transformed into a concentration-time curve Ct(t). Here the MRI signal change depends on the residue function R(t), which describes the fraction of contrast-agent present in the vasculature at time t, and the arterial supply from the feeding artery, which serves as a critical reference for calculating hemodynamic parameters. A parametric convolution method by Mouridsen et al.(2014) then relates the residue function to the distribution of vascular transit times, and thus to CTH. *Oxygen availability, represents the predicted maximum amout of oxygen provided by the circulation. Modified from Jespersen and Østergaard et al. (2012.

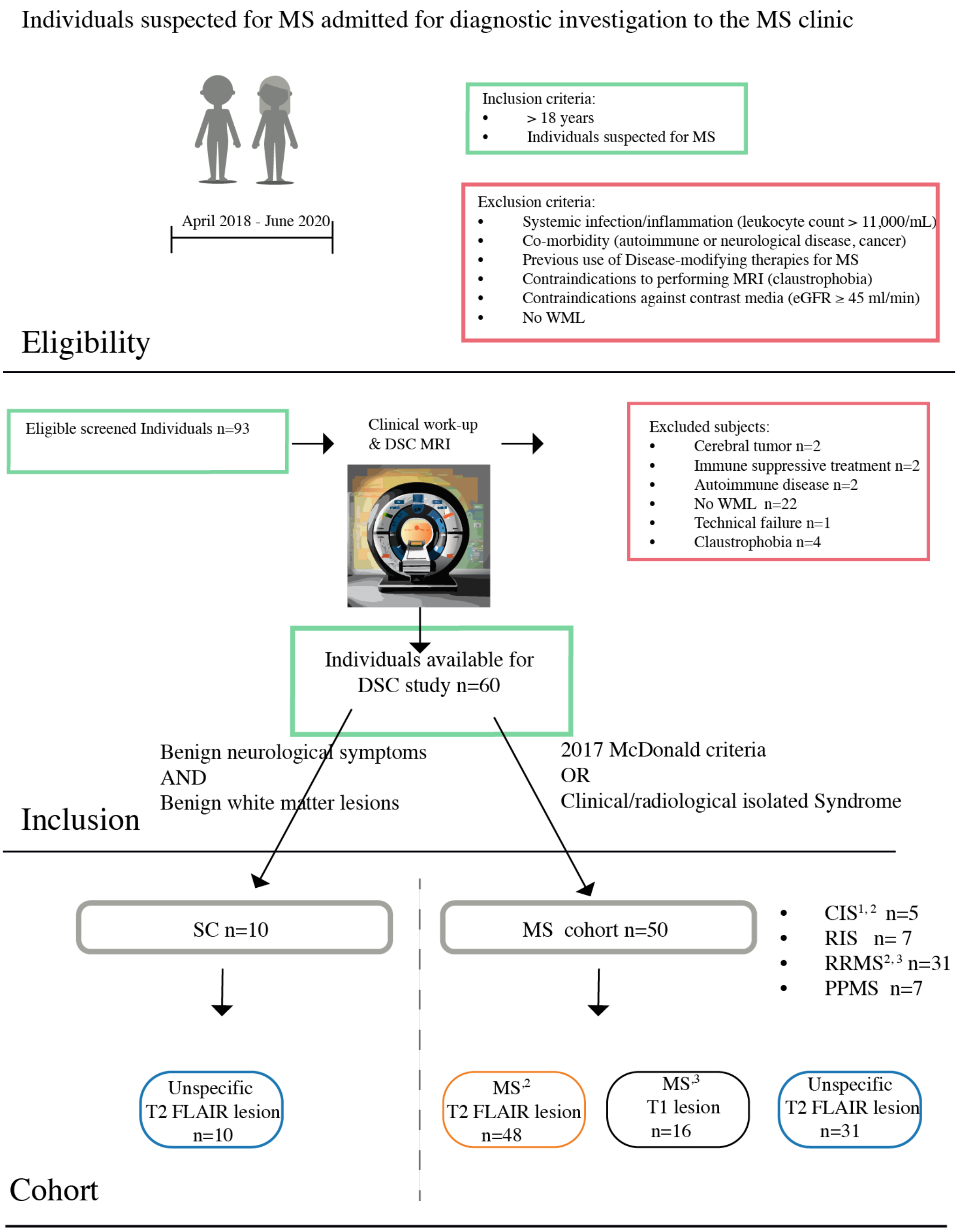

Figure S2. Flowchart of recruitment and inclusion procedure
1. Two (n=2) individuals classified with CIS converted to Manifest MS approximately 6 Months after recruitment.
2. Two (n=2) patients presented with MS T2-FLAIR lesions that were too small for analysis.
3. One (n=1) patient presented with a T1 lesion that was too small for analysis
Abbr. MS= Multiple sclerosis. WML= White matter lesion, eGFR=estimated glomerular filtration rate, SC= Symptomatic healthy control, n= number, CIS=Clinical Isolated Syndrome, RIS=Radiologic Isolated Syndrome, RRMS=Relapsing-Remitting MS, PPMS=Primary-Progressive MS.

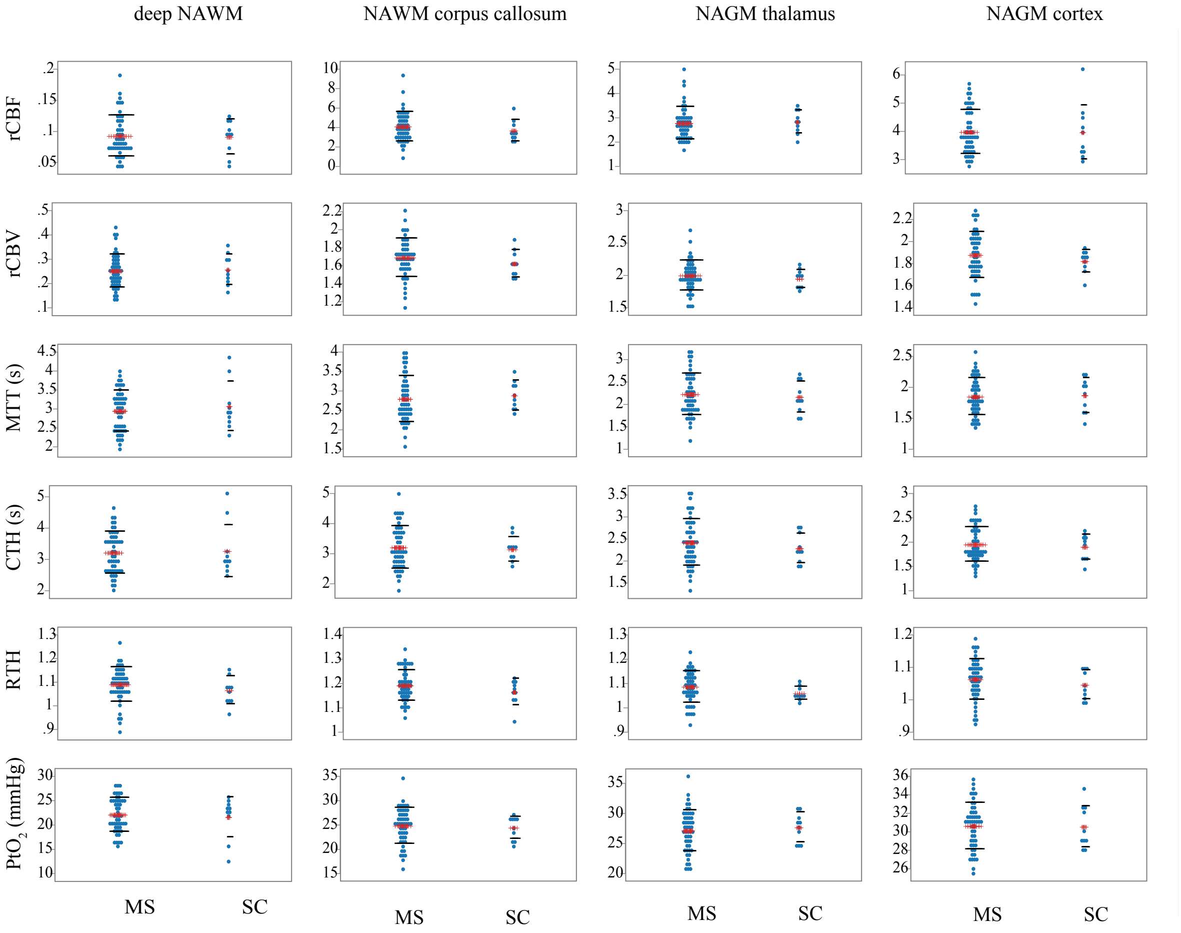

Figure S3. Unaltered perfusion in MS normal-appearing tissue.
Dot plots show mean perfusion (red cross) with standard deviation (dashed line) by MS and SC. Each dot represents one subject.
Data are based on DSC results extracted from MS (n=50) vs SC (n=10). Multivariate regression analysis, controlling for age and sex, was used to test the hypothesis that there was no difference between MS and SC. A two-sided p-value less than 0.05 was considered significant.
Abbr. SC= Symptomatic healthy control, n = number, NAGM= Normal appearing gray matter, NAWM= Normal appearing white matter, relative cerebral blood volume (rCBV), relative cerebral blood flow (rCBF), mean transit time (MTT),
capillary transit-time heterogeneity (CTH), relative transit-time heterogeneity (RTH), and tissue oxygen tension (PtO2).

Table S1 Paraclinical profile of MS and SC

Parameters	No (n)	MS	SC	P-value
CSF oligoclonal bands				0.001
No %(n)		22% (11)	90% (9)	
Yes %(n)		76% (38)	0% (0)	
Missing %(n)		2% (1)	10% (1)	
CSF cell count (cells / µL) mean (sd)	48/9	8 (8)	4(1)	0.16
CSF IgG index mean (sd)	48/8	0.9 (0.4)	0.5 (0.1)	0.04
IgG Index $_{elevated}$ [a]				0.020
No %(n)		38% (19)	70% (7)	
Yes %(n)		58% (29)	10% (1)	
Missing %(n)		4% (2)	20% (2)	
Albumin quotient (QAlb) elevated[b]				0.35
No %(n)		84% (42)	80% (8)	
Yes %(n)		10% (5)	0% (0)	
Missing %(n)		6% (3)	20% (2)	
CSF-to-serum albumin ratio mean (sd)	47/8	4.9 (2.6)	5.4 (2.4)	0.62
Blood hemogobin (mmol/L) mean (sd)	47/10	8.71 (0.98)	8.67 (0.64)	0.90
Blood leucocyte count (10^9 cells /L) mean (sd)	47/10	6.90 (2.17)	5.92 (1.41)	0.18
Blood lymphocyte count (10^9 cells /L) mean (sd)	46/9	1.83 (0.69)	1.88 (0.38)	0.82
MS T2-FLAIR lesion volume (ml)[c] mean (sd)	48/0	2.47 (3.93)		
CIS mean (sd)	4	0.92 (1.31)	0	
RIS mean (sd)	7	1.30 (1.92)	0	
RRMS mean (sd)	30	1.86 (2.41)	0	
PPMS mean (sd)	7	7.13 (7.61)	0	

Abbr.: Disease stage CIS=Clinical Isolated Syndrome, RIS=Radiologic Isolated Syndrome, RRMS=Relapsing-Remitting MS, PPMS=Primary-Progressive MS, CSF= Cerebrospinal Fluid, IgG= immunoglobulin G

a.The cut off for elevated IgG index was 0.67.

b.The ratio was normal if < 6.8 in patients below 45 years and < 10.2 in older patients

c. Lesion load based on manually outlined lesion masks co-registered to perfusion maps

Table S2

Test of between MS vs SC contrasts

Parameter	nMS/nSC	MS T2 lesion	Unspecific T2 lesion $_{SC}$	OR_{crude}	[95% Cl] $_{crude}$	p-value$_{crude}$	$OR_{adj.}$	[95% CI]$_{adj.}$	p-value$_{adj.}$
rCBV	48/10	1.21 (0.39)	0.98 (0.36)	1.320	[0.9, 1.8]	0.076	1.40	[0.9, 2.0]	0.077
rCBF	48/10	1.21 (0.44)	1.29 (0.47)	0.97	[0.8, 1.1]	0.62	0.95	[0.7,1.1]	0.518
MTT s	48/10	3.17 (0.69)	2.41 (0.71)	5.58**	[1.6, 19.8]	0.008	4.49*	[1.2, 17.3]	0.022
CTH s	48/10	3.34 (0.72)	2.55 (0.77)	5.52**	[1.5, 19.8]	0.009	4.45*	[1.2, 16.5]	0.026
RTH	48/10	1.06 (0.08)	1.07 (0.08)	0.83	[0.3, 2.2]	0.78	0.93	[0.4, 2.5]	0.827
PTO2	48/10	21.26 (4.01)	25.88 (4.98)	0,7634*	[0.6, 0.9]	0.002	0,789*	[0.6, 1.0]	0.018

Test of within MS subject contrasts

Parameter	nMS	MS T2 lesion	Unspecific T2 lesion $_{MS}$	p-value
rCBV	31	1.19 (0.30)	1.00 (0.28)	0.001
rCBF	31	1.23 (0.41)	1.19 (0.65)	0.05
MTT s	31	3.16 (0.78)	2.65 (0.73)	<0.001
CTH s	31	3.31 (0.78)	2.81 (0.84)	0.001
RTH	31	1.06 (0.07)	1.06(0.015)	0.58
PTO2	31	21.47 (4.38)	24.17 (4.79)	0.002

Test of within SC subject contrasts

Parameter	nSC	Unspecific T2 lesion $_{SC}$	NAWM control region$_{SC}$	p-value
CBV (AU)[a]	10	0.24 (0.04)	0.26 (0.06)	0.43
CBF (AU)[a]	10	0.11(0.03)	0.09(0.03)	0.11
MTT s	10	2.41 (0.71)	3.06 (0.66)	0.002
CTH s	10	2.55 (0.77)	3.26 (0.83)	0.001
RTH	10	1.07 (0.08)	1.06 (0.06)	0.847
PTO2	10	25.88 (4.98)	21.54 (4.14)	0.035

Parameter	nMS	Unspecific T2 lesion $_{MS}$	NAWM control region$_{MS}$	p-value
CBV (AU)[a]	31	0.25 (0.08)	0.25 (0.07)	0.94
CBF (AU)[a]	31	0.1 (0.06)	0.09 (0.03)	0.124
MTT (s)	31	2.65 (0.73)	2.98 (0.54)	0.005
CTH (s)	31	2.81 (0.84)	3.27 (0.67)	0.001
RTH	31	1.06(0.08)	1.1(0.07)	0.024
PTO2 (mmHg)	31	24.17 (4.79)	21.69 (3.46)	0.001

Parameter	nMS	MS T2 lesion	NAWM control region$_{MS}$	p-value
CBV (AU)[a]	48	0.30 (0.11)	0.25 (0.07)	0.001
CBF (AU)[a]	48	0.11 (0.40)	0.09 (0.03)	0.002
MTT (s)	48	3.17 (0.69)	2.93 (0.53)	0.003
CTH (s)	48	3.34 (0.72)	3.21 (0.64)	0.102
RTH	48	1.06 (0.08)	1.09 (0.07)	0.001
PTO2 (mmHg)	48	21.26 (4.01)	22.04 (3.35)	0.078

Note: a=CBF and CBV estimates are presented as arbitrary units (AU), while comparisons between lesions and NAWM are based on comparisions between normalized estimates (rCBF and rCBV).
DSC data expressed as mean and standard deviation (sd).

OR_{Crude} / 95% CI_{crude}: The influence of MS on DSC data (response variable), expressed by odds ratio different to one with 95% CI and p-values.

$OR_{adj.}$/ 95% $CI_{adj.}$: The influence of MS on DSC data (response variable) with adjustment for age and sex.

Table S3

Test of between MS subject contrasts: with (w) vs. without(w/o.) BBB leakage)

Parameter	n $_{w/oBBBleak}$ /n $_{w/BBBleak}$	MS T2 lesion $_{w/o.BBBleak}$	MS T2 lesion $_{w/BBBleak}$	OR_{crude}	[95% CI]$_{crude}$	p-value$_{crude}$	$OR_{adj.}$	[95% CI]$_{adj.}$	p-value$_{adj.}$
rCBV	16/32	1.26 (0.33)	1.19 (0.42)	1.53	[0.3, 7.0]	0.59	1.20	[0.23, 6.21]	0.83
rCBF	16/32	1.38 (0.48)	1.13 (0.40)	3.84	[0.8, 17.9]	0.06	2.56	[0.52, 12.53]	0.25
MTT s	16/32	2.98 (0.47)	3.26 (0.77)	0.53	[0.2, 1.4]	0.19	0.47	[0.7, 1.35]	0.14
CTH s	16/32	3.05 (0.48)	3.48 (0.78)	0.38	[0.1, 1.0]	0.06	0.31*	[0.1, 0.97]	0.04
RTH	16/32	1.03 (0.07)	1.08 (0.08)	0.41	[0.2, 1.2]	0.05	0.38*	[0.15, 0.96]	0.04
PTO2	16/32	22.71 (2.78)	20.54 (4.36)	1.16	[1.0, 1.4]	0.07	1.2	[0.99, 1.45]	0.06

Test of within MS subject contrasts (w. BBB leakage)

Parameter	nMS	MS T2 lesion	MS T1 lesion	p-value
rCBV	16	1.26(0.32)	1.23 (0.37)	0.80
rCBF	16	1.38(0.48)	1.25 (0.40)	0.38
MTT s	16	2.98(0.47)	2.94 (0.79)	0.83
CTH s	16	3.05(0.50)	2.77 (0.84)	0.19
RTH	16	1.02(0.04)	0.87 (0.09)	<0.01
PTO2	16	22.71(2.78)	23.64 (4.36)	0.41

Parameter	nMS	MS T1 lesion	NAWM control region	p-value
CBV (AU)[a]	16	0.29 (0.08)	0.24(0.06)	0.03
CBF (AU)[a]	16	0.11(0.03)	0.09(0.03)	0.03
MTT s	16	2.94 (0.79)	2.86 (0.51)	0.61
CTH s	16	2.77 (0.84)	3.07 (0.58)	0.1
RTH	16	0.87 (0.09)	1.07 (0.07)	<0.01
PTO2	16	23.64 (4.36)	22.61 (3.07)	0.27

Note: a= CBF and CBV estimates are presented as arbitrary units (AU), while comparisons between lesions and NAWM are based on normalized estimates (rCBF and rCBV).
DSC data expressed as mean and standard deviation (sd).

OR_{Crude} / 95% CI_{crude}: The influence of BBB leakage on DSC data (response variable), expressed by odds ratio different to one with 95% CI and p-values.

$OR_{adj.}$/ 95% $CI_{adj.}$: The influence of BBB leakage on DSC data (response variable) with adjustment for age and sex.

Table S4 (A). T2 lesion DSC metrics by disease course

	CIS	RIS	RRMS	PPMS
	n=4	n=7	n=30	n=7
rCBV	0.93 (0.19)	1.01 (0.20)	1.29 (0.43)	1.27 (0.29)
rCBF	1.26 (0.53)	1.05 (0.16)	1.27 (0.50)	1.11 (0.27)
**MTT s**	2.40 (0.56)	2.96 (0.53)	3.20 (0.59)	3.69 (0.90)
**CTH s**	2.50 (0.45)	3.10 (0.64)	3.39 (0.59)	3.82 (1.02)
**RTH**	1.07 (0.09)	1.05 (0.08)	1.07 (0.08)	1.03 (0.08)
**PTO2**	25.99 (3.17)	22.31 (3.30)	21.03 (3.38)	18.51 (5.44)

Note: DSC data expressed as mean and standard deviation (sd).

Supplementary Table S4 (B) Regression coefficents for predicting change in DSC meashurments

	rCBF			rCBV			MTT			CTH			RTH			PtO2		
	beta	CI	p-value	beta	CI	p-value	beta	CI	p-value	beta	CI	p-value	beta	CI	p-value	beta	CI	p-value
Sex	0.08	[-0.19 , 0.35]	0.57	0.02	[-0.22 , 0.25]	0.89	-0.5	[-0.87, -0.13]	0.01	-0.6	[-0.98, -0.23]	0.01	-0.27	[-0.76, 0.26]	0.266	3.19	[1.07, 5.33]	0.01
Age	-0.01	[-0.03 ,0.01]	0.11	-0.01	[-0.01, 0.01]	0.53	-0.01	[-0.03, 0.01]	0.5	-0.01	[-0.02, - 0.02]	0.73	0.01	[-0.02, 0.03]	0.756	0.03	[-0.08, 0.15]	0.59
T2 lesion load	-0.02	[-0.06 ,0.02]	0.31	-0.02	[-0.06, 0.01]	0.14	-0.01	[-0.06, 0.05]	0.89	0.01	[-0.05 ,0.05]	0.91	0.02	[-0.05 - 0.09]	0.577	-0.01	[-0.32 ,0.29]	0.93
CIS	1.63	[0.69 ,2.58]	0.001	1.09	[0.27, 1.91]	0.01	3.55	[2.24, 4.86]	0	3.7	[2.36, 5.03]	0	10.95	[9.22 ,12.7]	0	19.07	[11.55,26.59]	0
RIS	1.45	[0.61 ,2.30]	0.001	1.19	[0.45, 1.91]	0	3.95	[2.78,-5.12]	0	4.09	[2.95, 5.26]	0	10.7	[9.18 , 12.26]	0	16.45	[9.76, 23.15]	0
RRMS	1.60	[0.83 ,2.4]	0	1.45	[0.79, 2.12]	0	4.26	[3.20, -5.32]	0	4.49	[3.41,5.57]	0	10.93	[9.54 , 12,34]	0	14.7	[8.62, 20.78]	0
PPMS	1.74	[0.71 ,2.76]	0	1.62	[0.73, 2.52]	0	4.77	[3.34, 6.19]	0	4.83	[3.40 - 6.28]	0	10.36	[8.49 , 12.24]	0	12.42	[4.26,20.60]	0
The pairwise differences																		
CIS - RRMS	0.04	[-0.44, 0.52]	0.9	-0.36	[-0.78,0.06]	0.09	-0.71	[-1.37,0.04]	0.03*	-0.79	[-1.46,0.11]	0.02*	-0.01	[-0.87,0.88]	0.98	4.37	[0.56,8.19]	0.02*
CIS- PPMS	-0.1	[-0.72,0.52]	0.75	-0.53	[-1.07, 0.01]	0.05*	-1.21	[-2.08,0.35]	0.01*	-1.13	[-2.01,0.26]	0.01*	0.06	[-0.56,1.72]	0.31	6.64	[1.69,11.60]	0.01*
RRMS-PPMS	-0.14	[-0.62,0.35]	0.57	-0.17	[-0.60,0.25]	0.42	-0.51	[-1.18,0.17]	0.14	-0.34	[-1.03,0.34]	0.31	0.06	[-0.32,1.46]	0.2	2.27	[-1.60, 6.15]	0.25
Observations	48			48			48			48			48			48		
R-squared	0.902			0.924			0.970			0.972			0.995			0.978		

Note: Regression coefficients for predicing change in DSC meashuments 95% CI and p-values.

** $p<0.01$, * $p<0.05$
